# Evaluation of Self-Perceived Confidence and Competence in Oral Surgery among Final Year Undergraduate Students in Greece

**DOI:** 10.1055/s-0043-1771330

**Published:** 2023-12-29

**Authors:** Eliza Panagiotidou, Theodoros Lillis, Ioannis Fotopoulos, Demos Kalyvas, Nikolaos Dabarakis

**Affiliations:** 1 Department of Dentoalveolar Surgery, Implantology and Dental Anesthesiology, School of Dentistry, Aristotle University of Thessaloniki, Thessaloniki, Greece; 2 Department of Oral Surgery and Dental Anesthesia, School of Dentistry, National and Kapodistrian University of Athens, Athens, Greece

**Keywords:** oral surgery, dental undergraduate studies, confidence, competence, clinical skills, dentoalveolar surgery

## Abstract

**Objectives** Oral surgery is an integral part of dentistry that deals with the diagnosis and management of pathology of the mouth and jaws that requires surgical intervention. The aim of undergraduate studies in oral surgery is, upon graduation, to be confident and competent to treat without assistance surgical cases in the spectrum of general dentistry. This study evaluates the senior Greek dental students' self-confidence and self-perceived competence to undertake cases within the scope of oral surgery. Evaluation of clinical experience gathered during training and self-perceived confidence and competence in generic oral surgery skills is included.

**Materials and Methods** The present study was a questionnaire survey conducted during the academic year 2018–2019. The questionnaire comprised three sections. Section 1 included demographic data and four closed-ended questions concerning numerical data about procedures that they had already performed or observed, section 2 included four questions concerning their self-perceived competence to perform basic surgical techniques, and section 3 included 10 clinical case scenarios.

**Results** One hundred and twenty-seven students participated in the study. Among the basic surgical skills, students were most confident with suturing, and they were least confident with bone removal. Students from the Aristotle University of Thessaloniki (AUTH) tend to show higher level of confidence compared with students from the National and Kapodistrian University of Athens (NKUA) in most questions.

**Conclusion** Greek graduate dental students report moderate levels of self-confidence in oral surgery. A realistic approach in increasing self-confidence and competence in oral surgery would be the focus on preclinical training in generic elementary surgical skills, in combination with increased observational sessions of oral surgery procedures or outreach training.

## Introduction

Oral surgery is an integral part of dentistry that deals with the diagnosis and management of pathology of the mouth and jaws that requires surgical intervention. 1 The majority of European Union member states recognize oral surgery as a dental specialty, which comprises at least 3 more years of full-time training after completion of basic dental training. 2 However, the presence of recognized oral surgery specialists does not limit the range of activities of the general dental practitioners, who undertake many common oral surgical procedures. 2 3 4 The role of the specialist is considered to be a properly trained dentist who can carry out the more difficult and advanced procedures that may be beyond the abilities of the average general dental practitioner. 4 In this sense, a dental graduate must possess proper knowledge and skills to provide surgical management of the most common and basic cases in daily practice of general dentistry.

In 2010, the Association for Dental Education in Europe (ADEE) published the “Profile and competences for the graduating European dentist,” where the basic levels of professional behavior, theoretical knowledge, and clinical skills that a dentist must obtain upon graduation are described. 5 In this article, competences that fall within the scope of oral surgery are also described adequately. In the 2017 ADEE review 6 of these competences for the graduating European dentist, although oral surgery competences appear to have been limited, ADEE adopts and refers to the article of Macluskey et al, 7 in which a proposed curriculum for undergraduate oral surgery is more thoroughly described.

Competence is the ability to provide safe and reliable care on a consistent basis, which presupposes good theoretical knowledge and understanding of the subject along with sufficient clinical experience to treat, independently or without assistance, the clinical problems that arise. 8 The acquisition of competence is based on the gradual shifting of the treatment responsibility from the instructor to the learner, and it has been proposed that the learner passes through three stages, starting from “novice,” shifting to “beginner” and finally to “competent.” 9 10 In the “novice” stage, the learner is characterized by unconscious disability. In the “beginner” stage, although inexperienced, the learner is conscious of his or her impotence, while in the “competent” stage, the learner is able to independently perform treatments. After going through these three stages, the self-confidence of the learner increases.

Self-confidence is defined as an individual's own perception of performing a specific action. Although not necessarily representing the practitioner's actual ability, self-confidence strongly affects his or her decision to undertake a treatment or not. 11 Thus, as newly graduated dentists are required to perform procedures with limited clinical experience, boosting of self-confidence should be an integral aim of dental education. However, it is important to note that self-confidence should be mitigated by knowledge of personal limitations, weaknesses, and abilities so that the graduate dentist practices dentistry within safe limits. 12

The aim of the present study was to evaluate senior dental students' self-confidence and self-perceived competence to undertake cases within the scope of oral surgery. Evaluation of clinical experience gathered during training and self-perceived confidence and competence in generic oral surgery skills is included. Although similar studies have been conducted in several countries, to the best of our knowledge, this is the first to involve the two dental schools of Greece.

## Materials and Methods

The present study was a questionnaire survey conducted during the academic year 2018–2019, addressing the senior students of two dental schools of Greece, namely, Aristotle University of Thessaloniki (AUTH) and National and Kapodistrian University of Athens (NKUA). The study protocol was approved by the Institutional Ethical Committee and the candidate participants were informed that their participation was voluntary and anonymous.

All participants should have completed the first semester of the fifth year of study, and subsequently completed all theoretical courses and most of their clinical practice in the field of oral surgery. In both dental schools, theoretical courses begin in the third year and they are completed in the first semester of the fifth year. Moreover, preclinical practical teaching in suturing on models is also provided in both schools. Clinical training in oral surgery is provided during the fourth and fifth year of study. In the Dental School of AUTH, the students should complete at least 23 simple (nonsurgical) dental extractions and 2 cases that need incision and flap raising. In the Dental School of NKUA, the students should complete at least 25 dental extractions, while it is not mandatory to perform surgical cases. Moreover, in the Dental School of AUTH, the students should also observe at least 16 oral surgical operations performed by oral surgery residents or instructors, while in the Dental School of NKUA at least 2 such observations are mandatory.

The questionnaire of the survey was electronic and based on a similar one used in the study of Shah et al, 13 which was translated to the Greek language with some minor modifications to be harmonized with domestic data. Before its use in the present study, the questionnaire had been validated and piloted on the fourth-year dental students of the Dental School of the AUTH. The questionnaire comprised three sections. Section 1 included demographic data and the following four closed-ended questions concerning numerical data about the procedures that they had already performed or observed in the field of oral surgery during their clinical training:

- How many simple (nonsurgical) dental extractions have you already performed? (Answer: 1–5, 6–10, 11–15, 16–20, or >20.)
- How many surgical cases that needed incision and flap raising have you already performed? (Answer: 1–5, 6–10, 11–15, 16–20, or >20.)
- Have many surgical cases that needed osteotomy by using a surgical rotary device have you already performed? (Answer: 1–5, 6–10, 11–15, 16–20, or >20.)
- How many oral surgical operations performed by an experienced oral surgeon have you already observed? (Answer: 1–5, 6–10, 11–15, 16–20, or >20.)

Section 2 included the following four questions concerning their self-perceived competence to perform basic surgical techniques:

- How competent do you feel in performing incision and flap raising? (Answer: Not at all, Slightly, Moderately, Very, or Extremely.)
- How competent do you feel in performing tooth sectioning during extraction? (Answer: Not at all, Slightly, Moderately, Very, or Extremely.)
- How competent do you feel in performing osteotomy by using a surgical rotary device? (Answer: Not at all, Slightly, Moderately, Very, or Extremely.)
- How competent do you feel in performing wound suturing? (Answer: Not at all, Slightly, Moderately, Very, or Extremely.)

Section 3 included 10 clinical case scenarios ( Table 1 ) in which participants were asked to respond regarding their self-perceived competence to perform the necessary treatments with or without assistance, such as the following:

**Table 1:** Clinical case scenarios of section 3 of the questionnaire

Clinical case scenario	Photograph/radiograph
1. A 25-year-old male patient with clear medical history who presents for extraction of the erupted mandibular third molar	
2. A 60-year-old female patient with clear medical history who presents for multiple extractions and possible alveoloplasty to have complete dentures	
3. A 45-year-old female patient with clear medical history who presents for surgical extraction of retained roots of the mandibular second molar	
4. A 66-year-old female patient with osteoporosis and on oral alendronate medication that started 2 mo ago, who presents for extraction of the mandibular central incisors	
5. A 66-year-old female patient with osteoporosis, who has received to IV doses of alendronate in the past year and presents for extraction of a retained root of the maxillary canine	
6. A 58-year-old male patient with a history of coronary artery stenting who receives aspirin, clopidogrel, simvastatin, and metoprolol and presents for extraction of the maxillary lateral incisor	
7. A 62-year-old female patient with a history of atrial fibrillation who receives acenocoumarol (INR = 2.5) and presents for extraction of the retained root of the maxillary premolar	
8. A 37-year-old male patient with a clear medical history who presents for drainage of the vestibular abscess	
9. A 25-year-old male patient with a clear medical history, who presents for apicectomy of the maxillary central incisor	
10. A 62-year-old female patient with a clear medical history, who presents for excision of the fibrous hyperplasia of the buccal mucosa	

Abbreviations: INR, international normalized ratio; IV, intravenous.

- I feel competent to undertake the procedure, independently and without supervision or assistance from an instructor.
- I feel competent to undertake the procedure, but I may need some guidance or assistance from an instructor.
- I do not feel competent to undertake the procedure and I would refer the patient to an experienced dentist or oral surgeon.

Statistical analyses were performed by using SPSS (IBM SPSS Statistics 25.0). All answers of the questionnaire were categorical variables and they are presented as absolute and relative (%) frequencies. Comparison of frequencies between subgroups was performed with the chi-squared test, and when expected frequencies were lower than 5, comparison was performed with Fisher's exact test. Statistical significance was determined at *p* < 0.05 level.

## Results

One hundred and twenty-seven students participated in the study, representing over 60% of the final year students in two dental schools in Greece. Seventy-five (59.1%) were students of AUTH Dental School and 52 (40.95%) of NKUA Dental School. The mean age of the participants was 24.26 ± 1.6 years and the majority were females (74%).

Regarding answers of section 1 ( Table 2 ), most students had already performed over 20 simple (nonsurgical) dental extractions in both schools, without significant differences between them. Moreover, most students had already performed one to five surgical cases that needed incision and flap raising, although a significant difference was recorded between students from the two schools. Regarding osteotomy by using a surgical rotary device, most students had never performed any in both schools, without significant differences between them. Finally, great heterogeneity was recorded concerning the number of oral surgical operations that the students had observed, where the students of AUTH had observed marginally more operations than those of NKUA.

**Table 2:** Answers in questions of section 1 of the questionnaire

Question	Answers	Total *n* = 127 (%)	AUTH *n* = 75 (%)	NKUA *n* = 52 (%)	*p* -value
Q1. How many simple (nonsurgical) dental extractions have you already performed?	1–5 6–10 11–15 16–20 > 20	1 (0.8) 1 (0.8) 1 (0.8) 22 (17.3) 102 (80.3)	1 (1.3) 0 (0.0) 0 (0.0) 10 (13.3) 64 (85.3)	0 (0.0) 1 (1.9) 1 (1.9) 12 (23.1) 38 (73.1)	0.114
Q2. How many surgical cases that needed incision and flap raising have you already performed?	1–5 6–10 11–15 16–20 > 20	110 (86.6) 10 (7.9) 6 (4.7) 1 (0.8) 0 (0.0)	72 (96.0) 3 (4.0) 0 (0.0) 0 (0.0) 0 (0.0)	38 (73.1) 7 (13.5) 6 (11.5) 1 (1.9) 0 (0.0)	< 0.001
Q3. How many surgical cases that needed osteotomy by using surgical rotary device have you already performed?	1–5 6–10 11–15 16–20 > 20	39 (30.7) 40 (31.5) 34 (26.8) 13 (10.2) 1 (0.8)	21 (28.0) 22 (29.3) 22 (29.3) 10 (13.4) 0 (0.0)	18 (34.6) 18 (34.6) 12 (23.1) 3 (5.8) 1 (1.9)	0.366
Q4. How many oral surgical operations performed by an experienced oral surgeon have you already observed?	1–5 6–10 11–15 16–20 > 20	13 (10.2) 26 (20.5) 45 (35.4) 16 (12.6) 27 (21.3)	6 (8.0) 11 (14.7) 27 (36.0) 14 (18.7) 17 (22.7)	7 (13.5) 15 (28.8) 18 (34.6) 2 (3.8) 10 (19.2)	0.044

Abbreviations: AUTH, Aristotle University of Thessaloniki; NKUA, National and Kapodistrian University of Athens.

Regarding answers of section 2 ( Table 3 ), most students felt “slightly” to “moderately” competent to perform incision and flap raising, and the students of AUTH were feeling significantly more competent than those of NKUA. Similarly, most students felt “slightly” to “moderately” competent and low percentage of students felt “very” competent in performing tooth sectioning during extraction, and the students of AUTH were feeling significantly more competent than those of NKUA. Regarding performing an osteotomy using a surgical rotary device, most students were more or less equally divided in feeling “not at all,” “slightly,” or “moderately” competent to perform it in both schools, without significant differences between them. Finally, most students felt “moderately” to “very” competent and a considerable number of students felt “extremely” competent in performing wound suturing, although the students of NKUA were feeling significantly less competent to perform this task than those of AUTH.

**Table 3:** Answers in questions of section 2 of the questionnaire

Question	Answers	Total *n* = 127 (%)	AUTH *n* = 75 (%)	NKUA *n* = 52 (%)	*p* -value
Q1. How competent do you feel in performing incision and flap raising?	Not at all Slightly Moderately Very Extremely	15 (11.8) 40 (31.5) 53 (41.7) 17 (13.4) 2 (1.6)	7 (9.3) 17 (22.7) 38 (50.7) 12 (16.0) 1 (1.3)	8 (15.4) 23 (44.2) 15 (28.8) 5 (9.6) 1 (1.9)	0.027
Q2. How competent do you feel in performing tooth sectioning during extraction?	Not at all Slightly Moderately Very Extremely	9 (7.1) 42 (33.1) 46 (36.2) 25 (19.7) 5 (3.9)	8 (10.7) 21 (28.0) 32 (42.7) 13 (17.3) 1 (1.3)	1 (1.9) 21 (40.4) 14 (26.9) 12 (23.1) 4 (7.7)	0.031
Q3. How competent do you feel in performing osteotomy by using a surgical rotary device?	Not at all Slightly Moderately Very Extremely	39 (30.7) 40 (31.5) 34 (26.8) 13 (10.2) 1 (0.8)	21 (28.0) 22 (29.3) 22 (29.3) 10 (13.3) 0 (0.0)	18 (34.6) 18 (34.6) 12 (23.1) 3 (5.8) 1 (1.9)	0.366
Q4. How competent do you feel in performing wound suturing?	Not at all Slightly Moderately Very Extremely	2 (1.6) 14 (11.0) 26 (20.5) 60 (47.2) 25 (19.7)	2 (2.7) 3 (4.0) 11 (14.7) 43 (57.3) 16 (21.3)	0 (0.0) 11 (21.2) 15 (28.8) 17 (32.7) 9 (17.3)	0.002

Abbreviations: AUTH, Aristotle University of Thessaloniki; NKUA, National and Kapodistrian University of Athens.

Regarding answers of section 3 ( Table 4 ), more than one-third of the students felt competent to perform extraction of an erupted mandibular third molar, multiple dental extractions with minor alveoloplasty, or surgical extraction of the retained root (clinical cases 1, 2, and 3) independently. Approximately half of them would prefer to have an instructor's guidance or assistance while operating, and very few students said they would refer these cases to a specialist. Regarding the cases of dental extractions in patients on oral or intravenous (IV) bisphosphonate medication (clinical cases 4 and 5), about one-third of the students felt competent to undertake the cases independently, one-third believed that they would need an instructor's guidance to undertake the cases, and about one-third would refer those cases. A small shift toward the need for an instructor's guidance or referring was recorded in case of patients on IV bisphosphonate medication in comparison with the case of patient on oral bisphosphonate medication. Regarding the cases of dental extractions in patients on antithrombotic medication (clinical cases 6 and 7), answers were similar. While a little less than one-third of the students felt competent to undertake the case independently, more than one-third of the students felt that they would need an instructor's guidance to undertake the cases, and a little less than one-third of the students would refer the cases. No statistically significant differences were recorded in the answers between students of the two dental schools, concerning the above clinical case scenarios.

**Table 4:** Answers in clinical case scenarios of section 3 of the questionnaire

Clinical case scenario	Answers	Total *n* = 127 (%)	AUTH *n* = 75 (%)	NKUA *n* = 52 (%)	*p* -value
1. Extraction of the erupted mandibular third molar	a b c	44 (34.6) 73 (57.5) 10 (7.9)	32 (42.7) 37 (49.3) 6 (8.0)	12 (23.1) 36 (69.2) 4 (7.7)	0.056
2. Multiple extractions with minor alveoloplasty	a b c	40 (31.5) 73 (57.5) 14 (11.0)	26 (34.7) 38 (50.7) 11 (14.6)	14 (26.9) 35 (67.3) 3 (5.8)	0.118
3. Surgical extraction of the retained root	a b c	54 (42.5) 59 (46.5) 14 (11.0)	35 (46.7) 35 (46.7) 5 (6.6)	19 (36.5) 24 (46.2) 9 (17.3)	0.142
4. Extraction in a patient on oral bisphosphonate medication	a b c	48 (37.8) 39 (30.7) 40 (31.5)	24 (32.0) 24 (32.0) 27 (36.0)	24 (46.2) 15 (28.8) 13 (25.0)	0.234
5. Extraction in a patient on IV bisphosphonate medication	a b c	26 (20.5) 50 (39.4) 51 (40.2)	11 (14.7) 29 (38.7) 35 (46.6)	15 (28.8) 21 (40.4) 16 (30.8)	0.083
6. Extraction in patient on dual antiplatelet medication	a b c	35 (27.6) 60 (47.2) 32 (25.2)	19 (25.3) 39 (52.0) 17 (22.7)	16 (30.8) 21 (40.4) 15 (28.8)	0.434
7. Extraction in a patient on coumarin anticoagulant medication	a b c	35 (27.6) 58 (45.7) 34 (26.7)	22 (29.3) 33 (44.0) 20 (26.7)	13 (25.0) 25 (48.1) 14 (26.9)	0.851
8. Incision and drainage of the vestibular abscess	a b c	53 (41.7) 56 (44.1) 18 (14.2)	40 (53.3) 27 (36.0) 8 (10.7)	13 (25.0) 29 (55.8) 10 (19.2)	0.006
9. Apicectomy of the anterior tooth	a b c	7 (5.5) 16 (12.6) 104 (81.9)	6 (8.0) 14 (18.7) 55 (73.3)	1 (1.9) 2 (3.8) 49 (94.3)	0.008
10. Excisional biopsy of mucosal fibrous hyperplasia	a b c	15 (11.8) 45 (35.4) 67 (52.8)	12 (16.0) 33 (44.0) 30 (40.0)	3 (5.8) 12 (23.1) 37 (71.1)	0.002

Abbreviations: AUTH, Aristotle University of Thessaloniki; IV, intravenous; NKUA, National and Kapodistrian University of Athens.

Regarding incision and drainage of the dentoalveolar abscess (clinical case 8), most students felt competent to undertake the procedure, although approximately half of them would prefer to have an instructor's guidance or assistance while operating and a relatively low proportion of students said they would refer the case. Regarding apicectomy of the anterior maxillary tooth (clinical case 9), a vast majority of students would refer the case. Very few students said they would undertake the procedure independently or with an instructor's supervision or assistance. Finally, regarding excision of the mucosal fibrous hyperplasia (clinical case 10), approximately half of the students would refer the case, while a few students said they would undertake the procedure with an instructor's guidance or assistance, and even fewer students said they would undertake the procedure independently. Considering the above three cases, the students of AUTH, although fewer in number, were feeling significantly more competent to undertake these procedures than the students of NKUA.

## Discussion

Acquisition of clinical competence and self-confidence is a primary objective of the curricula in dentistry, although standards of undergraduate teaching and extent of clinical training largely defer among dental schools worldwide. Oral surgery is considered a discipline in which dental students have lower levels of self-confidence upon graduation in comparison with other disciplines of dentistry. 14 To the best of our knowledge, this is the first study to evaluate self-perceived confidence and competence in oral surgery among final year undergraduate dental students in Greece. In general, the results of the study indicate acceptable levels of self-confidence to undertake simpler oral surgical procedures and lower levels of self-confidence to undertake more difficult or complex cases, which may be considered an expected finding.

Achieving the competence to perform simple (nonsurgical) exodontia by using forceps and root elevators is considered a primary target of undergraduate training in oral surgery. 15 The majority of final-year Greek dental students had already approached the minimum mandatory dental extractions (>20), although this represents a relatively low number in comparison to other similar studies 16 17 18 19 20 21 reporting 20 to 60 dental extractions in the final year. However, a great heterogeneity in the total number of extractions (1–200) that the graduates finally perform is recorded in these studies. Although a minimum target number of dental extractions is usually applied in undergraduate training in oral surgery, it is considered difficult to define a universal number of cases that would ensure undergraduate students' competence and confidence, as some students feel capable after a small number of dental extractions, while others felt incompetent even after a large number of dental extractions. 22 23 Considering this, the achievement of learning objectives and the acquisition of surgical skills in all students may not be possible with the generalized quantification of the minimum number of cases. This could be achieved with the early identification of students with increased learning needs and the creation of a supportive environment with individualized assignment of appropriate cases.

The competence in surgical extractions or other more advanced oral surgical procedures is considered more difficult to be delivered due to limited undergraduate exposure. 15 16 These procedures prerequire adequate training in generic elementary surgical skills such as incision and flap raising, tooth sectioning, bone removal with surgical rotary devices, and wound suturing. The majority of Greek dental students reported low to moderate levels of self-confidence to perform incision and flap raising or tooth sectioning, lower levels of self-confidence to perform osteotomy, but high levels of self-confidence in suturing. These findings are probably due to the fact that most students had already undertaken one to five cases that needed incision, flap raising, and consequently wound suturing, while very small students had undertaken at least one case that needed osteotomy with a surgical rotary device. Moreover, preclinical training on models in both Greek dental schools included in the study includes only training in wound suturing and not in incisions, flap raising, or osteotomy. The finding of higher level of self-confidence in wound suturing compared with other generic elementary surgical skills is consistent in most similar studies. 13 19 20 24

The clinical cases of this study's questionnaire represent cases in which graduates are expected to demonstrate a sound theoretical knowledge and understanding of the subject, according to the curriculum proposals adopted by the ADEE. Furthermore, the first three scenarios represented cases in which graduates are expected to have adequate clinical experience to be competent to undertake them independently or without assistance, while the rest of the scenarios represent cases in which graduates may have only limited clinical/practical experience. A similar study including similar case scenarios was conducted by Shah et al 13 in 2015 on final-year dental undergraduate students at King's College London, from which the present study's questionnaire was obtained and modified with similar cases. In the present study, however, three additional cases have been added regarding the drainage of a dental abscess, performing an apicoectomy of the anterior tooth, and removing a fibrous hyperplasia. In general, students at King's College show significantly more confidence than students in Greek universities in almost all cases, probably due to the increased clinical exposure of the former (mean number of nonsurgical and surgical extractions performed was 64 and 9, respectively). Moreover, Shah et al 13 included more older age students (29–40 years) than our study, who generally report higher levels of self-confidence according to the same study.

In clinical case 1 of the present study, which may be considered a difficult extraction although it is nonsurgical, half of the students report that they would need some guidance or assistance from an instructor to undertake the procedure. This finding is consistent with previous studies, 18 19 which also report that final-year students feel more confident to undertake a single rooted tooth extraction than a posterior multirooted tooth. This indicates that generally undergraduate students may need more training in posterior multirooted teeth nonsurgical extractions than in anterior single rooted tooth extractions. Considering clinical cases 2 and 3, almost half of the students also report that they would need some guidance or assistance from an instructor to undertake the procedure. This finding may be the result of students' limited experience and lower self-confidence in generic elementary surgical skills.

The following four case scenarios of the present study represent cases of nonsurgical extractions in patients on common medication (antithrombotics or bisphosphonates). The medical complexity of these cases demands sound theoretical knowledge on the management of medically compromised patients. In general, it seems that Greek dental students feel more confident to perform dental extractions in patients on antithrombotic medication than on bisphosphonate therapy, which is also reported in the study of Shah et al 13 conducted at King's College. However, students at King's College report much higher self-confidence to independently treat patients on antithrombotic medication, while Greek students report higher self-confidence to treat patients on IV bisphosphonates. These differences may be explained by differences not only in both clinical exposures but also in the extent of theoretical courses on proper management of medically compromised dental patients.

The last three case scenarios of the present study represent the cases in which the students have limited clinical experience and exposure. In total, Greek students feel more confident to perform incision and drainage of the dentoalveolar abscess than to perform an apicectomy of the anterior tooth or excisional biopsy of the mucosal fibrous hyperplasia. In these case scenarios, the students of AUTH seem to feel significantly more confident to undertake these cases than the students of NKUA. Interestingly, the students of AUTH in comparison with those of NKUA had observed significantly more operations performed by an experienced oral surgeon, but they had performed significantly lesser cases that needed incision and flap raising. Thus, it may be assumed that when the number of cases to perform is small and cannot provide competency and confidence, regular observation and assistance in corresponding procedures performed by a skilled instructor can significantly help gain confidence and improve skills. The important role of observing surgical operations performed by seniors and outreach in teaching of oral surgery has also been proposed by previous studies. 19 24 25

Admittedly the present study bears the limitation of evaluating the self-perceived confidence of the students. Although it is considered that there is a positive correlation between reported confidence and clinical experience, 26 self-assessment of surgical skills may be inaccurate, 27 particularly in those who perform poorly. 28 Moreover, overconfident graduates can put their patients at risk when carrying out surgical procedures without actual competency. Thus, objective assessment tools for surgical skills should be adopted to evaluate the graduates' competency in daily dental practice.

## Conclusion

Greek graduate dental students report moderate levels of self-confidence in oral surgery. Although there are several factors affecting the confidence of dental students, the structure of the training program can be considered to have an important role. Traditional teaching methods for increasing self-confidence would suggest increasing the clinical experience by increasing the number and complexity of clinical cases undertaken by the students. However, this would be difficult to apply in the undergraduate training of oral surgery, as the students' clinical training should be balanced between several other disciplines of dentistry. Thus, another approach in increasing self-confidence and actual competence in oral surgery would be to focus on observational sessions of oral surgery procedures or outreach training, together with achieving proficiency in generic elementary surgical skills. These skills are part of a complete surgical procedure and, in combination with the growing clinical experience, will allow the general dentist to undertake surgeries of varying difficulty over the years.
